# Cell deaths: Involvement in the pathogenesis and intervention therapy of COVID-19

**DOI:** 10.1038/s41392-022-01043-6

**Published:** 2022-06-13

**Authors:** Xue Li, Ziqi Zhang, Zhenling Wang, Pedro Gutiérrez-Castrellón, Huashan Shi

**Affiliations:** 1 Department of Biotherapy, State Key Laboratory of Biotherapy and Cancer Center, West China Hospital, Sichuan University, No. 17, Block 3, Southern Renmin Road, Chengdu, Sichuan 610041 People’s Republic of China; 2 Laboratory of Aging Research and Cancer Drug Target, State Key Laboratory of Biotherapy, National Clinical Research Center for Geriatrics, West China Hospital, Sichuan University, No. 17, Block 3, Southern Renmin Road, Chengdu, Sichuan 610041 People’s Republic of China; 3 State Key Laboratory of Biotherapy and Cancer Center, West China Hospital, Sichuan University, and Collaborative Innovation Center of Biotherapy, Ke Yuan 4th Road, Gao Peng Street, Chengdu, Sichuan 610041 People’s Republic of China; 4 Center for Translational Research on Health Science, Hospital General Dr. Manuel Gea Gonzalez. Ministry of Health, Calz. Tlalpan 4800, Col. Secc. XVI, 14080, Mexico city, Mexico

**Keywords:** Infection, Infectious diseases

## Abstract

The current pandemic of coronavirus disease 2019 (COVID-19) caused by severe acute respiratory syndrome coronavirus 2 (SARS-CoV-2) infection has dramatically influenced various aspects of the world. It is urgent to thoroughly study pathology and underlying mechanisms for developing effective strategies to prevent and treat this threatening disease. It is universally acknowledged that cell death and cell autophagy are essential and crucial to maintaining host homeostasis and participating in disease pathogenesis. At present, more than twenty different types of cell death have been discovered, some parts of which have been fully understood, whereas some of which need more investigation. Increasing studies have indicated that cell death and cell autophagy caused by coronavirus might play an important role in virus infection and pathogenicity. However, the knowledge of the interactions and related mechanisms of SARS-CoV-2 between cell death and cell autophagy lacks systematic elucidation. Therefore, in this review, we comprehensively delineate how SARS-CoV-2 manipulates diverse cell death (including apoptosis, necroptosis, pyroptosis, ferroptosis, and NETosis) and cell autophagy for itself benefits, which is simultaneously involved in the occurrence and progression of COVID-19, aiming to provide a reasonable basis for the existing interventions and further development of novel therapies.

## Introduction

Coronavirus disease 2019 (COVID-19) pandemic caused by severe acute respiratory syndrome coronavirus 2 (SARS-CoV-2)^1^ is disastrous to public health, the global economy, and society as a whole human. As of 8 June 2022, the cumulative number of the World Health Organization (WHO) reported cases is 530,896,347 with 6,301,020 deaths (for constantly updated information, see: https://covid19.who.int). SARS-CoV-2 infects the host cells by binding with angiotensin-converting enzyme-2 (ACE2)^2^ or CD147^3^ receptors on the cell membrane and being assisted with transmembrane serine protease 2 (TMPRSS2) and protease Furin.^4^ Besides, SARS-CoV-2 also utilizes phosphatidylinositol 3-phosphate 5-kinase (PIKfyve), two-pore channel subtype 2 (TPC2), cathepsin L (CTSL) -mediated endocytosis for entry.^5^ Once entering into the host cell, SARS-CoV-2 replicates RNAs and expresses proteins, assembles into complete viral particles, and eventually releases virions, concurrently leading to cell death and cellular content release.^6^ The ACE2 receptor is widely expressed in the lungs, kidneys, gastrointestinal system, cardiovascular system, central nervous system, etc.^7^ From mild to severe, the clinical manifestations of COVID-19 include asymptomatic infection, pulmonary and extra-pulmonary symptoms (such as fever, fatigue, cough, diarrhea, nausea, loss of taste and smell, etc.), acute respiratory distress syndrome (ARDS), acute kidney injury (AKI), multiple organ failure, and even death.^8^

It is generally acknowledged that cell death and cell autophagy are fundamental cellular activities, playing crucial roles in maintaining homeostasis and disease pathogenesis. Over the past decades, more than twenty types of cell death have been identified by the Nomenclature Committee on Cell Death (NCCD),^9^ to which apoptosis, necroptosis, and pyroptosis are paid more attention. Previous studies have suggested that viruses can delicately regulate cell death through multiple mechanisms in different types of cells. A growing body of evidence indicates that these processes are considered double-edged swords during virus infection.^10,11^ On the one hand, the replication and spread of the virus are impeded due to the clearance of infected cells via cell death. However, on the other hand, dysregulated cell death causes uncontrol cell damage and disordered immune response. Meanwhile, viruses can hijack cell autophagy to be advantageous for replication niches, immune evade, and extracellular release.^12^ Currently, the relationship between SARS-CoV-2, cell death, and cell autophagy is not well established, but to some extent, could be referred to previous findings of SARS-CoV and Middle East respiratory syndrome coronavirus (MERS-CoV).^13,14^ Herein, we discuss cell death and cell autophagy involved in the pathogenesis and intervention therapies of COVID-19, providing a potential direction for the repurposing of old drugs and the development of novel treatments.

## SARS-CoV-2: structure and characteristics

Coronaviruses are classified into four genera, namely alpha-, beta-, gamma-, and delta-coronavirus, with the genomes ranging from 26 to 32 kb. SARS-CoV-2 is an enveloped, positive-sense single-stranded RNA (+ssRNA) with a length of 29.9 kb. This genomic RNA consists of four structural regions (which encode Spike (S), Envelope (E), Membrane (M), Nucleocapsid (N) protein), and thirteen open reading frames (ORFs) (which encode nonstructural proteins (NSP1-16) and accessory proteins).^15^ All of them are involved in virus entry, translation of replication machinery, virus replication, translation of structure proteins, virion assembly, and virus release.^16^ Furthermore, the next-generation sequencing presented that SARS-CoV-2 shares 79.4% homology to SARS-CoV and 50% to MERS‐CoV.^16^

## Apoptosis and SARS-CoV-2

### Apoptosis core machinery

Apoptosis is a common programmed cell death (PCD) with immunological silence studied by J. Kerr first via electron microscopy in 1972.^17^ The morphological alterations of apoptosis are characterized by cell shrinkage, condensation and fragmentation of chromatin, and forming of apoptotic bodies. Apoptosis is vital in maintaining cellular homeostasis and regulating physiological and pathological events.^18,19^ Apoptosis can be triggered mainly by the extrinsic and intrinsic pathways. Interacting with the extracellular death ligands, including Fas ligand (FasL or CD95), tumor necrosis factor-alpha (TNF-α), TNF-related apoptosis-inducing ligand (TRAIL), and interferon-gamma (IFN-γ), the death receptors (DRs) immediately recruit Fas-associated protein with death domain (FADD) or TNF receptor 1-associated death domain protein (TRADD) and pro-cysteine-aspartic protease (procaspase)−8/10 to assemble the death-inducing signaling complex (DISC), causing cleavage of procaspase-8, and then, active caspase-8 directly cleaves procaspase-3/6/7 to induce cell apoptosis. Meanwhile, a truncated form of the BH3 interacting domain death agonist (BID) processed by active caspase-8 can translocate into mitochondria, leading to B-cell lymphoma-2 (Bcl-2)-associated X (BAX)/Bcl-2 homologous killer (BAK) mediated-mitochondrial outer membrane permeabilization (MOMP) for releasing apoptotic factors cytochrome c (cyt c) into the cytoplasm. Afterward, the apoptosome composed of cyt c, the apoptotic protease activating factor-1 (Apaf-1), and caspase-9 activates executioner caspases-3/6/7. The intrinsic pathways stimulated by DNA damage and stress can directly cause mitochondrial damage and MOMP to release cyt c. The other subsequent events have been described as above.^20^

### Apoptosis inhibition by SARS-CoV-2

As of current evidence, it is shown that apoptosis is an important event during SARS-CoV-2 infection. SARS-CoV-2 infection can, directly and indirectly, upregulate the expression of cellular FADD-like interleukin-1 (IL-1)-converting enzyme-inhibitory protein (c-FLIP), which modulate death effector domains to suppress caspase-8/10 activation.^21^ The upregulation of miRNA-155 at the beginning of virus infection was reported by the small RNA profiling analysis of SARS-CoV-2 infected human cell lines^22^ for reducing the expression of forkhead transcription factor O 3a (FoXO3a) with the ability to reverse the inhibition of c-FLIP.^23^ It is acknowledged that the nuclear factor kappa B (NF-κB) pathway can upregulate transcriptional expression of some important apoptosis inhibitors, such as c-FLIP, X-linked inhibitor of apoptosis proteins (XIAPs), and Bcl-2.^24^ Activation of the (NF-κB) pathway has been identified upon SARS-CoV-2 infection.^25,26^ Besides, SARS-CoV-2 proteins including NSP13 and ORF9c were indicated to interact with the NF-κB pathway according to proteomic analyses.^15^ SARS-CoV-2 S protein was also reported to activate Toll-like receptor 2 (TLR2)-dependent NF-κB signaling pathway in human and mouse lung epithelial cells.^27^ Hence, it is reasonable speculation that SARS-CoV-2 would utilize apoptosis inhibition to avoid elimination, so as to obtain enough time and place for replication at the early stage.

### Apoptosis induction by SARS-CoV-2

Accumulative studies have demonstrated that SARS-CoV-2 proteins greatly participate in apoptosis induction in various aspects. Similar to the previous findings of SARS-CoV ORF3a,^28,29^ SARS-CoV-2 ORF3a is also identified as a viroporin, with the ability to form an ion channel on the cell membrane to disturb intracellular homeostasis, which is responsible for apoptosis and promote virus release.^30,31^ Another study showed that SARS-CoV-2 ORF3a could efficiently induce apoptosis by directly cleaving and activating caspase-8 for the extrinsic pathway and cross-talk to the intrinsic pathway via tBID, leading to the cyt c release and caspase-9 activation.^32^ Besides, SAR-CoV-2 ORF7b was reported to promote the expression of TNF-α and induce TNF-α-dependent apoptosis in HEK293T cells and Vero E6 cells.^33^

**Fig. 1:**
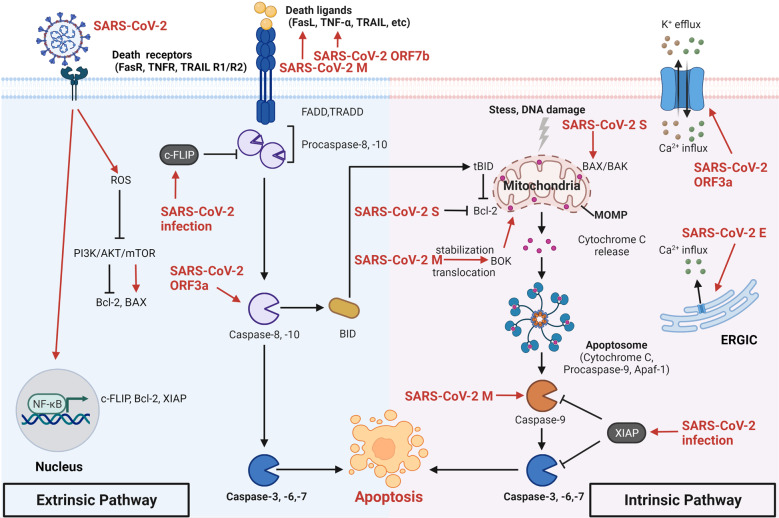
The mechanisms of SARS-CoV-2 regulating cell apoptosis. Activated death receptors recruit and cleave caspase-8/10, subsequently activating caspase-3/6/7 to induce extrinsic cell apoptosis. Stress- and DNA damage-stimulated cytochrome c directly cleaves caspase-9 to activate caspase-3/6/7 for intrinsic cell apoptosis. And tBID caused by caspase-8 also induces intrinsic apoptotic way. SARS-CoV-2 infection-upregulated c-FLIP and NF-κB inhibit apoptosis in infected cells. In contrast, SARS-CoV-2 ORF7b- and M protein-upregulated death ligands and SARS-CoV-2 ORF3a-activated caspase-8 promote extrinsic apoptosis. And SARS-CoV-2 S protein regulates Bcl-2 and BAX to induce the intrinsic apoptotic pathway. SARS-CoV-2 M protein-stabilized BOK and -directly activated caspase-9 both induce intrinsic apoptosis. Besides, cellular ion imbalance caused by SARS-CoV-2 ORF3a and E protein is involved in infected cell apoptosis. SARS-CoV-2 severe acute respiratory syndrome coronavirus 2, S spike, E envelope, M membrane, ORF open reading frame, NF-κB nuclear factor kappa B, c-FLIP cellular Fas-associated protein with death domain-like interleukin-1-converting enzyme-inhibitory protein, Bcl-2 B-cell lymphoma-2, XIAP X-linked inhibitor of apoptosis protein, ROS reactive oxygen species, PI3K phosphoinositide 3-kinase, AKT protein kinase B, mTOR mammalian target of rapamycin, TNF-α tumor necrosis factor-alpha, TRAIL TNF-related apoptosis-inducing ligand, FADD Fas-associated protein with death domain, TRADD TNF receptor 1-associated death domain protein, Caspase cysteine-aspartic protease, tBID truncated BH3 interacting domain death agonist, BOK Bcl-2 ovarian killer, BAX Bcl-2-associated X, BAK Bcl-2 homologous killer, MOMP mitochondrial outer membrane permeabilization, Apaf-1 apoptotic protease activating factor-1, ERGIC endoplasmic reticulum-Golgi intermediate compartment. Created with BioRender

Apart from the accessory proteins, the structural proteins also can trigger apoptosis by multiple mechanisms. SARS-CoV-2 E protein is sub-located in the endoplasmic reticulum (ER)-Golgi intermediate compartment (ERGIC) and serves as an ion channel,^34^ which is in accordance with the previous study of SARS-CoV E protein.^35^ Inhibition of E protein with a channel inhibitor BE-33 reduced both viral load and cell damage of SARS-CoV-2 infection in the hACE2 mouse model. Furthermore, the SARS-CoV-2 M protein regulates cell apoptosis in two ways. Firstly, SARS-CoV-2 M protein directly inhibits the ubiquitination of Bcl-2 ovarian killer (BOK) (a pro-apoptotic protein^36^) and promotes its translocating to the mitochondria, leading to H292 cell apoptosis via the intrinsic way.^37^ In addition, the pulmonary damage caused by M protein overexpression can be alleviated by the knockdown of BOK. Secondly, M protein suppresses 3-phosphoinositide-dependent protein kinase 1 (PDK1)-protein kinase B (PBK)/AKT axis, thereby decreasing the activity of its substrates, including forkhead transcription factor (FKHRL1) and apoptosis signal-regulating kinase (ASK).^38^ The reduction in phosphorylated FKHRL1 boosts its translocation to the nucleus for upregulating FasL expression on the membrane for the extrinsic apoptosis. Meanwhile, the inhibition of caspase-9 is reversed by dephosphorylated ASK, triggering the intrinsic apoptosis. Besides, SARS-CoV-2 S protein can activate reactive oxygen species (ROS)-inhibited phosphoinositide 3-kinase (PI3K)/AKT/ mammalian target of rapamycin (mTOR) pathways,^39^ subsequently inducing autophagy-triggered apoptosis and inflammation.^40–42^ It is noteworthy that SARS-CoV-2 has weaker pro-apoptotic activity than SARS-CoV,^32,43^ which may partially explain the more cases of asymptomatic patients with widespread viral transmission. Mutations of SARS-CoV-2 ORF3a attenuate cytosolic form-related apoptosis, whereas SARS-CoV ORF3a could well leverage this way. In addition, previous studies have shown that SARS-CoV can promote cell apoptosis in various other ways, including ORF3a- and ORF7a-induced p38 mitogen-activated protein kinase (MAPK) activation,^44,45^ ORF3a- and ORF6-induced ER stress,^46,47^ and N protein-upregulated JNK and p38 MAPK activities,^48^ all of which should be further investigated on SARS-CoV-2. The underlying mechanisms that SARS-CoV-2 regulates cell apoptosis are summarized in Fig. 1.

### Apoptosis during SARS-CoV-2 infection

Increasing evidence has demonstrated that apoptosis is involved in the pathogenesis of COVID-19 might be due to massive cell damage and tissue injury, such as in the lung, kidney, liver, pancreas, nervous system, immune system, etc. Apoptosis was detected by TdT-mediated dUTP nick end labeling (TUNEL) staining in lung samples from human patients and the non-human primate (NHP) model of SARS-CoV-2 infection which is responsible for accelerating the progression of ARDS.^42^ Further study showed that the intrinsic and extrinsic pathways triggered apoptosis in various cell types, including alveolar type 1 and 2 cells (AT1s and AT2s), vascular endothelial cells (ECs), macrophages, and T cells in the infected NHP lungs. A number of studies on SARS-CoV-2-induced apoptosis in respiratory epithelial cells and ECs align with the above findings.^49–52^ A large number of the renal tubular epithelial cells also occur apoptosis, leading to AKI in the mouse model with SARS-CoV-2 infection.^53^ In addition, SARS-CoV-2 induced β cell apoptosis via the JNK-MAPK apoptotic pathway, resulting in attenuated insulin production and secretion and exacerbation of diabetes.^54^ In addition, the phosphoproteomics data of human islets revealed that apoptosis-related signaling pathways were upregulated during SARS-CoV-2 infection.

Dysregulation of immune response is considered as a very important characteristic in SARS-CoV-2 infection.^25,55^ Lymphopenia, manifested with reduced absolute numbers of CD4^+^ and CD8^+^ T cells,^56^ has been related to the severity of COVID-19 patients.^57^ Two studies found that FasL expression on CD4^+^ and CD8^+^ T cells in COVID-19 patients is significantly higher than that in healthy controls, contributing to cell apoptosis,^58^ which correlates with lymphopenia and severe condition.^59^ Meanwhile, apoptosis-related caspase activation was also observed in T cells of infected patients.^60^ Moreover, the single-cell RNA sequencing (scRNA-seq) analysis of CD3^+^ T cells from COVID-19 patients indicted the upregulation of cell death-related genes.^61^ In this study, dysfunctional mitochondria with irregular shapes and incomplete cristae accompanied by cyt c release were exhibited by confocal and transmission electron microscopy, linking to apoptosis in T cells, which could be rescued by caspases inhibitor. Besides, surface FasL expression on B cells in the lymph nodes (LNs) of COVID-19 indicated SARS-CoV-2-promoted B-cell apoptosis.^62^

The decreased proportion of dendritic cells (DCs) in COVID-19 patients is another immune feature, which also correlates with the disease severity of SARS-CoV-2 infection.^56,63,64^ Damaged mitochondria and caspase-3 activation-dependent apoptosis were found in monocyte-derived macrophages (MDMs) and DCs, which could be prevented via anti-IFNγ treatment.^65^ Another study further indicated that IFNγ-induced macrophage cell death is mediated by caspase-8.^66^ On the one hand, active caspase-8 directly processes caspase-3 for the extrinsic cell apoptosis; on the other hand, it downregulates the expression of Bcl-2 to promote mitochondrial-driven cell apoptosis. Interestingly, upregulated inducible nitric oxide synthase (iNOS) promotes the cleavage of caspase-8 and instability of anti-apoptotic protein myeloid cell leukemia 1 (Mcl-1) to reinforce cell apoptosis. Besides, the conditional-independence network analysis suggested that apoptotic gene signatures (including BRCA2, CASP3, CASP8, BID, BAK1, and XBP1) significantly increased in plasmacytoid DCs (pDCs) from COVID-19, which is related to low-frequency pDCs and disease severity.^67^ Another scRNA-seq data was in accordance with this study.^68^ Generally speaking, pDCs are the major subset of secreted type I IFN (including IFN-α and IFN-β) in response to viral infection.^69^

### Apoptosis-targeted therapies

As described above, two completely opposite approaches should be considered for the treatment according to the different stages of SARS-CoV-2 infection. The impeded apoptosis of host cells is advantageous for viral replication at the early stage, which emerges as a potential target for COVID-19 treatment. Therefore, inducing apoptosis cascade via blocking the suppressor (such as c-FLIP and NF-κB) might be beneficial for the host. In this regard, the histone deacetylase inhibitors (such as LBH589) with inhibitory effect on c-FLIP can be applied to COVID-19 patients.^70^ Many anti-inflammatory and antioxidative agents, such as phillyrin,^71^ dexamethasone,^72^ hydroxychloroquine,^73^ macrolide antibiotics,^74^ and N-acetylcysteine,^75^ have adverse effects on SARS-CoV-2 replication partially due to inhibition of the NF-κB pathway.^76^ It should be noticed that inhibiting NF-κB would contribute to significant side effects and adverse reactions for the reason that suppression of immune response.

In the later stage, SARS-CoV-2 strongly induces apoptosis in various host cells, resulting in a large amount of tissue damage and loss of function, which accelerates the development of disease and even death. Thereby, impeding apoptotic pathways by blocking DRs signaling and caspase cascade, has been proposed as a promising strategy to reduce viral widespread and alleviate disease progression. Emricasan, an irreversible pan-caspase inhibitor, was reported to protect human cortical neural progenitors from caspase-3-induced cell death during Zika virus infection,^77^ which is considered as a possible drug for COVID-19 patients. However, the clinical trial on emricasan for mild-COVID-19 treatment has been terminated for the reason of difficulty in recruiting patients (NCT04803227). Unfortunately, the majority of caspase inhibitors are only utilized in cell and animal experiments, thus requiring further experiments explored in clinical trials.

It has been well established that TNF-α plays a vital role in the pathogenesis of infectious diseases, inflammatory processes, and malignant tumors.^78^ Increasing studies have shown that elevated circulating levels of TNF-α are positively associated with disease severity and death of COVID-19 patients.^79–81^ A recent study displayed that co-treatment of TNF-α and IFN-γ could substantially induce cell death, including apoptosis in PMA-differentiated macrophage-like THP-1 cells.^82^ Thus, targeting TNF-α and its receptor is a promising therapy for SARS-CoV-2 infection. Infliximab is a mouse-human chimeric monoclonal antibody to effectively block TNF-α and prevent its binding to receptors, which has obtained the US Food and Drug Administration (FDA)-approval for treatment with rheumatoid arthritis, psoriatic arthritis, ankylosing spondylitis, and plaque psoriasis. A single-arm phase II trial indicated that Infliximab treatment decreased pathological inflammatory cytokines in severe and critical hospitalized COVID-19 patients (NCT04425538).^83^ Other clinical trials of Infliximab are under investigation (NCT04922827, NCT05220280, NCT04593940,^84^ NCT05273242). Adalimumab, a humanized anti-TNF-α monoclonal antibody, has been utilized with remdesivir and dexamethasone for severe COVID-19 patients in a randomized controlled trial. Nevertheless, no positive results were observed in mortality, mechanical ventilation requirement, hospital and ICU stay duration, and even radiologic changes.^85^ Additionally, Etanercept serves as a TNF-α/β inhibitor and is undergoing clinical trial (NCT05080218). Intriguingly, it is found that anti-TNF-α treatment would have negative effects on antibody response in response to SARS-CoV-2 infection.^86^

## Necroptosis and SARS-CoV-2

### Necroptosis core machinery

Necroptosis is a form of PCD and caspase-independent lytic cell death, which was first observed by J. Yuan in 2005.^87^ Morphological changes of necroptosis include early loss of plasma membrane integrity, organelle swelling, and nuclear condensation. Binding with death ligands (including TNF-α, FasL, and IFN-γ), the DRs recruit TRADD, FADD, caspase-8, receptor-interacting protein kinase 1 (RIPK1) and RIPK3 to assemble the necrosome. Inactivation of caspase-8 caused autophosphorylation of RIPK1-RIPK3, which further phosphorylate mixed lineage kinase domain-like protein (MLKL). pMLKL oligomerize to form pores in the cytomembrane leading to cell death and release of damage-associated molecular patterns (DAMPs) and cytokines that can stimulate immune responses.^88,89^ The TLRs signaling pathway and viral RNA-Z-DNA/RNA binding protein-1 (ZBP1, also known as DAI) axis also can directly interact and activate RIPK3 to induce necroptosis.^90,91^

### Necroptosis interplay with SARS-CoV-2 and -targeted therapies

**Fig. 2:**
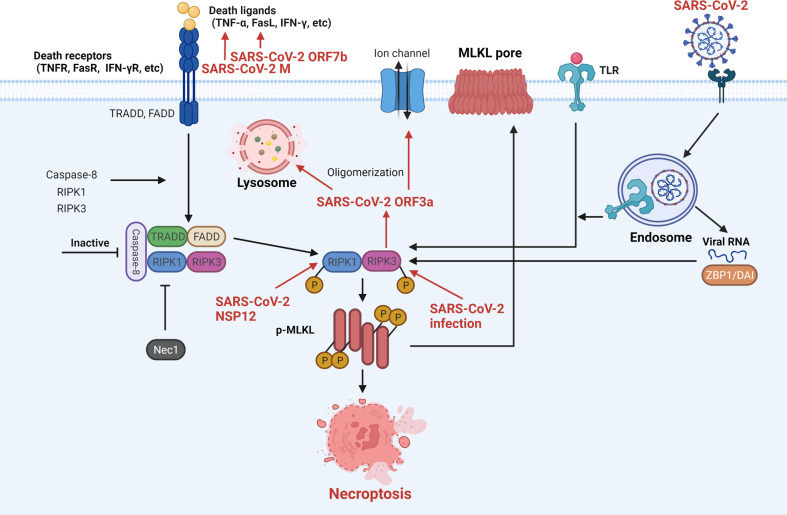
The interaction of SARS-CoV-2 and cell necroptosis. Activated death receptors phosphorylate RIPK1 and RIPK3 in the absence of active caspase-8, and consecutively phosphorylate MLKL to form pores in the cell membrane, eventually leading to cell necroptosis. SARS-CoV-2 ORF7b and M protein-upregulated death ligands, SARS-CoV-2 NSP12-activated RIPK1, and SARS-CoV-2-related RIPK3 activation all promote necroptosis in infected cells. Meanwhile, the SARS-CoV-2-stimulated TLRs pathway and viral ZBP1 axis also directly activate RIPK3 to induce necroptosis. Besides, RIPK3-induced SARS-CoV-2 ORF3a oligomerization enhances its functions. SARS-CoV-2 severe acute respiratory syndrome coronavirus 2, M membrane, ORF open reading frame, NSP nonstructural protein, IFN-γ interferon-gamma, TRADD TNF receptor 1-associated death domain protein, FADD Fas-associated protein with death domain, Caspase cysteine-aspartic protease, RIPK receptor-interacting protein kinase, Nec1 necrostatin-1, MLKL mixed lineage kinase domain-like protein, TLR Toll-like receptor, ZBP1/DAI Z-DNA/RNA binding protein-1. Created with BioRender

The interaction between the host cell necroptosis and SARS-CoV-2 has not yet been totally elucidated. In a recent study, RIPK1 activation was found in human COVID-19 lung samples.^92^ Treatment with Necrostatin-1(Nec1, a known RIPK1 inhibitor) reduced viral load, inflammation, and damage in cells and mouse models infected with SARS-CoV-2. It is demonstrated that necroptosis plays an important role in the pathogenesis of COVID-19. Furthermore, SARS-CoV-2 NSP12 can directly interact with and promote RIPK1 activation. Another study showed the fluorescence staining of pMLKL pores on the cytomembrane in SARS-CoV-2-infected cells and human postmortem lungs in another study.^93^ SARS-CoV-2 can induce RIPK3 to promote cell necroptosis, which is significantly attenuated by RIPK3 inhibitors. Interestingly, a previous study reported that RIPK3-induced SARS-CoV ORF3a oligomerization augments membrane insertion and ion channel function of 3a, finally resulting in multimodal cell death in a human lung cell line (including necroptosis).^94^ The interaction of SARS-CoV-2 with cell necroptosis is concluded in Fig. 2.

Although blocking the virus replication via cell death, necroptosis conversely accelerates viral widespread and the release of cellular contents by cell rupture. It was reported that the serum RIPK3 levels are higher in severe patients than in mild ones, suggesting that RIPK3 may be involved in the progression of COVID-19 pneumonia to ARDS.^95,96^ Thus, targeting the components of the necroptotic pathway might be a therapeutic approach for COVID-19.^97^ Both the RNA-sequencing data and proteome-wide data analysis revealed that RIPK1 is a potential drug target for COVID-19 patients.^98,99^ The FDA-approved drug primidone has the property to reduce RIPK1-mediated necroptosis in vitro and TNFα-induced inflammation in vivo,^100^ which may be beneficial to patients with COVID-19. The drugs targeting RIPK3 (GSK872) and MLKL (necrosulfonamide) have not yet been further investigated in COVID-19.

## Pyroptosis and SARS-CoV-2

### Pyroptosis core machinery

Pyroptosis is a lytic form of PCD driven by inflammasome activation, which was first termed by B. Cookson in 2001.^101^ In morphology, cell swelling and rupture, DNA condensation and fragmentation, and membrane pores formation are observed in pyroptosis. Pyroptosis plays a crucial role in inflammation and immunity,^102^ thus involved in the development of diverse diseases.^103^ Pyroptosis can be induced by mainly two inflammasome pathways: the caspase-1-dependent canonical pathway and the caspase-4/5/11-dependent noncanonical pathway. In the priming phase, pathogen-associated molecular patterns (PAMPs) and damage-associated molecular patterns (DAMPs) are recognized by various receptors (such as TLRs, TNFR, and IL-1R1), resulting in NF-κB activation to promote pro-inflammatory cytokines production including nucleotide-binding oligomerization domain-like receptor containing pyrin domain 3 (NLRP3), procaspase-1, pro-IL-1β, and pro-IL-18. In the activation phase, PAMPs- and DAMPs-activated NLRP3 recruits the apoptosis-associated speck-like protein containing a CARD (ASC) and procaspase-1 to form inflammasome specks generating active caspase-1 that further cleaves pro-IL-1β, pro-IL-18, and gasdermin-D (GSDMD). GSDMD-N inserts into cytomembrane to form GSDMD pores, leading to cell pyroptosis and contents liberation (such as IL-1β, IL18, IL-6, TNFα, IL-8, DAMPs, etc).^104^ In addition, lipopolysaccharide (LPS)-induced activation of caspase-4/5/11 can directly cleave GSDMD to form pores and trigger cell pyroptosis.

### Pyroptosis regulation by SARS-CoV-2 and host system

It is well known that the activation of inflammasomes is the rate-limiting step of cell pyroptosis, which can be affected by a wide range of factors, such as toxins, viruses, bacteria,^105^ ATP change,^106^ ion imbalance,^107,108^ ROS production,^109^ mitochondrial dysfunction,^110^ lysosomal destruction,^111^ etc. Growing studies have indicated that various proteins encoded by SARS-CoV-2 regulate inflammasome activity through multiple mechanisms. As described above, SARS-CoV-2 ORF3a and E protein are identified as viroporins for ion disorder. SARS-CoV-2 ORF3a induces K^+^ efflux and Ca^2+^ influx^112^ whereas E protein only promotes Ca^2+^ influx,^34^ resulting in a decrease in cellular K^+^ level and an increase in Ca^2+^ level. Previous studies showed that ion imbalance not only directly activates NLRP3 inflammasome but also augments the generation of mitochondrial ROS. Furthermore, SARS-CoV-2 ORF3a works as a bridge for promoting the interaction of never in mitosis gene a (NIMA)-related kinase 7 (NEK7) and NLRP3, leading to IL-1β release in lung epithelial cell line A549.^112^ The NEK7 is an essential mediator of NLRP3 activation.^113^ In addition, lysosomal dysfunction caused by SARS-CoV-2 ORF3a also has been reported, manifesting K^+^ efflux, Ca^2+^ influx, and cathepsins leakage.^114^ Apart from ORF3a, SARS-CoV-2 N protein directly interacts with NLRP3 protein, further promoting the interaction of NLRP3 with ASC and assembly of the inflammasome.^115^ Besides, SARS-CoV-2 NSP6 impairs lysosomal acidification via directly interacting with ATP6AP1 (a vacuolar ATPase proton pump component), leading to autophagic flux stagnation, which facilitates NLRP3 inflammasome activation and pyroptosis in lung epithelial cell lines.^116,117^ Intriguingly, in turn, SARS-CoV-2 also possesses the ability to diminish host cell pyroptosis, which might be utilized to strive for replication time at the early stage. SARS-CoV-2 NSP1 and NSP13 both can directly inhibit NLRP3 inflammasome-induced active caspase-1 in macrophage-like THP-1 cells.^118^ NSP5 antagonizes pyroptosis by cleaving GSDMD at Q193-G194 junction into two inactive fragments with ineffective pores formation.^119^ Moreover, SARS-CoV-2 N protein protects GSDMD from caspase-1 cleavage via occupying the linker region of GSDMD in infected human monocytes.^120^

The human ACE2 receptor is widely expressed in many cell types, whose primary function is converting angiotensin II (Ang II) to angiotensin 1–7.^121^ Due to ACE2 being occupied mainly by SARS-CoV-2, Ang II degradation is attenuated, leading to Ang II accumulation. Hyperactivation of Ang II-angiotensin 1 receptor (AT1R) signaling induces mitochondrial dysfunction with ROS production, resulting in NLRP3 inflammasome-related pyroptosis,^122,123^ which is further confirmed in line with recent results of hematopoietic stem cells (HSCs) and endothelial progenitor cells (EPCs) damaged by SARS-CoV-2 infection.^124^

A number of studies indicated that the complement system is activated during SARS-CoV-2 infection, releasing C3a, C5a, and membrane attack complex (MAC), which is mediated by mannan-binding lectin (MBL) - MBL-associated serine protease (MASP).^125–127^ For example, SARS-CoV-2 N protein activates the MASP2-mediated complement pathway whereas S protein activates the alternative complement pathway.^128^ Previous studies have demonstrated that complement cascade can augment the activation of NLRP3 inflammasome and cell pyroptosis. The C3a-mediated ATP efflux initiates P2 x 7, an ATP-gated ion channel for K^+^ efflux.^129^ The C5a upregulates the expression of double-stranded RNA-dependent protein kinase (PKR),^130^ an important regulator of NLRP3 inflammasome activation.^131^ Additionally, the MAC can insert into the cytomembrane to form transmembrane pores for Ca^2+^ influx.^132^

**Fig. 3:**
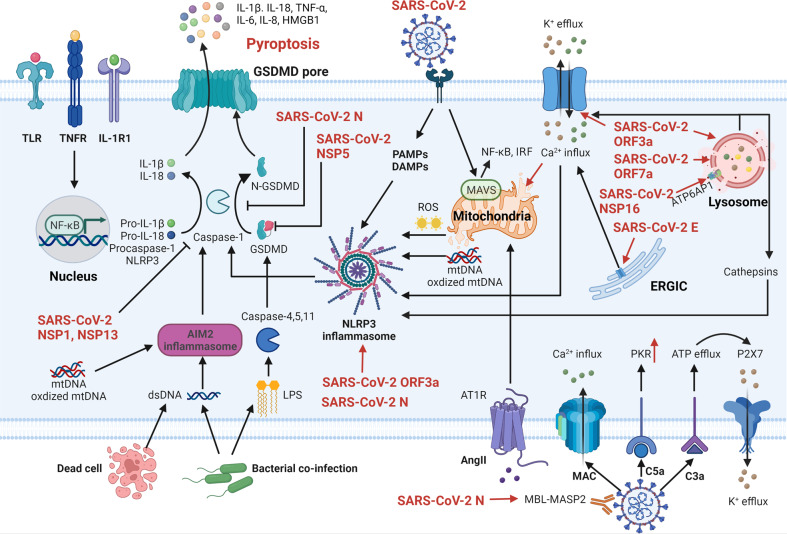
The sophisticated associations of SARS-CoV-2, host system, and cell pyroptosis. Activated TLRs, TNFR, and IL-1R1 pathways induce NF-κB activation to promote pro-inflammatory cytokines production including pro-IL-1β, pro-IL-18, procaspase-1, and NLRP3. The PAMPs and DAMPs caused by SARS-CoV-2 immediately activate the NLRP3 inflammasome to cleave procaspase-1 that subsequently cleaves pro-IL-1β and pro-IL-18. Meanwhile, caspase-1-cleaved GSDMD inserts into the membrane to form pores, leading to cell pyroptosis and contents release. SARS-CoV-2 N protein, NSP5, NSP1, and NSP13 inhibit infected cell pyroptosis via impeding the caspase-1 or GSDMD. On the contrary, more commonly, SARS-CoV-2 promotes cell pyroptosis in various ways. SARS-CoV-2 ORF3a and N protein directly activate the NLRP3 inflammasome. The ion imbalance caused by SARS-CoV-2 ORF3a and E protein and lysosomal dysfunction caused by SARS-CoV-2 ORF3a, ORF7a, and NSP16 both promote the NLRP3 inflammasome activation. In addition, the MAVS pathway activated by SARS-CoV-2 infection causes mitochondria damage and the activation of NF-κB and IRF. Besides, SARS-CoV-2-related ROS production and mtDNA release also activate the NLRP3 inflammasome. Meanwhile, dsDNA and mtDNA-induced AIM2 inflammasome and LPS-induced caspase-4/5/11 also induce cell pyroptosis. From the perspective of the host, the Ang II-AT1R axis and the MBL-MASP-mediated complement system are involved in infected cell pyroptosis. SARS-CoV-2 severe acute respiratory syndrome coronavirus 2, N nucleocapsid, E envelope, ORF open reading frame, NSP nonstructural protein, TLR Toll-like receptor, TNFR tumor necrosis factor receptor, IL-1R1 interleukin-1 receptor 1, NF-κB nuclear factor kappa B, IRF transcription factors interferon regulatory factor, TNF-α tumor necrosis factor-alpha, HMGB1 high mobility group box 1, GSDMD gasdermin-D, Caspase cysteine-aspartic protease, AIM2 absent in melanoma, dsDNA double-stranded DNA, LPS lipopolysaccharide, PAMPs pathogen-associated molecular patterns, DAMPs damage-associated molecular patterns, NLRP3 nucleotide-binding oligomerization domain-like receptor containing pyrin domain 3, ROS reactive oxygen species, mtDNA mitochondrial DNA, Ang II angiotensin II, AT1R Ang II-angiotensin 1 receptor, MBL-MASP mannan-binding lectin (MBL)-MBL-associated serine protease, MAC membrane attack complex, PKR double-stranded RNA-dependent protein kinase, ERGIC endoplasmic reticulum-Golgi intermediate compartment. Created with BioRender

Furthermore, absent in melanoma 2 (AIM2) inflammasome also activates caspase-1 and promotes cell pyroptosis, which was detected in SARS-CoV-2-infected human monocytes.^133^ It has been previously shown that cytoplasmic double-stranded DNA, such as mitochondrial DNA (mtDNA), oxidized mtDNA,^134,135^ and cell-free DNA (cfDNA)^136^ can directly trigger AIM2 inflammasome-mediated pyroptosis. During SARS-COV-2 infection, massive dysfunctional mitochondria would release mtDNA and oxidized mtDNA (induced by a large amount of ROS),^137^ whereas dead cell releases cfDNA.^138^ The complex associations among SARS-CoV-2, host system and cell pyroptosis are summarized in Fig. 3.

### Pyroptosis during SARS-CoV-2 infection

Mounting evidence has implied that inflammasome activation and cell pyroptosis play pivotal roles in the pathogenesis of COVID-19. For one thing, this process causes massive cell damage and tissue injury. For another, it releases excessive inflammatory cytokines and DAPMs, termed as cytokine storm, strongly accelerating the development of COVID-19. Activated NLRP3 and AIM2 inflammasome-dependent pyroptosis were observed via fluorescence staining in infected circulating monocytes and lung tissue-resident macrophages from COVID-19 patients,^139^ in which NLRP3 activation was further confirmed through virus stimulation in other studies.^133,140^ In another study, compared to healthy controls, cell pyroptosis and cytokine storm occurred in CD163^+^ pro-inflammatory macrophages in lung samples from severe COVID-19 patients.^141^ SARS-CoV-2 pseudovirus-treated macrophage-like THP-1 cells also activated caspase-1, cleaved GSDMD, and secreted cytokines. Apart from the classical pathway, SARS-CoV-2-derived ssRNA can facilitate TLR8-dependent NLRP3 inflammasome activation and cytokines release in human macrophages without pyroptosis.^142^ Surprisingly, HSCs and EPCs are also damaged by NLRP3 inflammasome-mediated pyroptosis during SARS-CoV-2 infection,^143,144^ destroying hematopoietic and coagulation functions. Furthermore, it has been reported that SARS-CoV-2 triggers NLRP3 inflammasome-mediated pyroptosis in the endothelium,^145^ thus leading to endothelial dysfunction in the respiratory and myocardial system.^146,147^

Importantly, SARS-CoV-2 infection initiates cytokine storm, in which inflammasome-dependent cell pyroptosis has been proven as the very important inducer, manifested by elevated levels of pro-inflammatory cytokines and chemokines,^148^ such as IL-6, IL-1β, TNFα, IL-8, IL-10, CXCL10, MCP1, which strongly correlated with the COVID-19 severity and mortality.^79,149,150^ In this context, these inflammatory mediators strongly promote cell migration and activation, further emphasizing the inflammatory environment and tissue damage. Meanwhile, multiple types of cell death enhance epithelial and endothelial cell permeability, providing convenience for cell migration and fluid leakage, aggravating the progression of acute lung injury (ALI) and ARDS.^151,152^ It has been stated that SARS-CoV-2 spreads into the cardiovascular system via the circulatory or lymphatic system,^153^ as does in the lung, inducing uncontrolled cell deaths and triggering the pro-inflammatory cascade,^154,155^ resulting in myocarditis, loss of cardiac functions, and vascular diseases.^156,157^

### Pyroptosis-targeted therapies

As of current evidence, inflammasome-mediated cell pyroptosis dramatically affects the development and progression of COVID-19, supporting it as a promising therapeutic target, particularly in severe cases. Two strategies could be under consideration: inhibition of the basic components (such as NLRP3 inflammasome, GSDMD, and cytokines) and inhibition of the enhancer (such as ion imbalance, ROS production, and Ang II-AT1R axis) of cell pyroptosis. A recent study showed that targeting NLRP3 inflammasome by gene knockout or selective inhibitor MCC950 ameliorated overactive inflammatory response and pathology of lung in mice models with SARS-CoV-2 infection,^158^ as does in the previous study of influenza A.^159^ A multicenter, randomized, controlled phase II trial of DFV890 (a selective NLRP3 inflammasome inhibitor) treatment for COVID-19 with pneumonia and impaired respiratory function exhibited negative results (NCT04382053). There is no significant difference between the two groups: standard of care (SoC) plus DFV890 treatment and only SoC. A phase II clinical trial of selective NLRP3 inflammasome inhibitor Dapansutrile for moderate COVID-19 with early cytokine release syndrome is recruiting (NCT04540120). Colchicine, a microtubule polymerization inhibitor, has been identified with the ability to inhibit NLRP3 inflammasome^160,161^ and investigated in clinical trials for COVID-19.^162^ A randomized, double-blind, placebo-controlled clinical trial in moderate to severe COVID-19 patients suggested that colchicine reduces oxygen supplement and hospitalization.^163^ Besides, colchicine was reported to decrease the rate of hospitalization and death of non-hospitalized patients with COVID-19 in a randomized, double-blind, placebo-controlled phase III trial (NCT04322682).^164^ However, the large-scale clinical trials of colchicine are disappointing. A randomized, controlled phase II/III trial in hospitalized COVID-19 patients indicated that colchicine could not reduce mechanical ventilation requirement, length of hospital stay, and 28-day mortality (NCT04381936),^165^ in line with results of another clinical trial (NCT04328480).^166^ Thus, the efficacies of colchicine by the single or combined treatment should be further studied.

GSDMD-dependent pores work as the executor of cell pyroptosis and cytokine release, implying the treatment possibility for inhibiting GSDMD. The FDA-approved disulfiram (DSF) ^167^ and dimethyl fumarate (DMF) ^168^ both have the property of inhibiting GSDMD pore formation in cells and animal models. It is noteworthy that DSF also can suppress virus replication by inhibiting the polyprotein proteases (including main protease (M^pro^) and papain-like protease (PL^pro^)), NSP13, and NSP14 of SARS-CoV-2.^169^ Meanwhile, DMF exhibits antiviral activity by upregulating nuclear factor erythroid-2 related factor 2 (NRF2) antioxidant gene expression^170^ in SARS-CoV-2-infected cells.^171^ A retrospective study showed lower SARS-CoV-2 infection risk in alcohol use disorder patients with DSF treatment than without DSF treatment (hazard ratio (HR) 0.66).^172^ A randomized, double-blind, placebo-controlled phase II trial to evaluate the efficacy and safety of DSF in moderate COVID-19 patients has been completed but no result has been published yet (NCT04594343). Other clinical trials of DSF (NCT04485130) and DMF (NCT04381936, NCT04792567) in COVID-19 are under evaluation.

Cell pyroptosis causes numerous inflammatory cytokines release, among which IL-1β can in turn boost inflammasome activation in a positive feedback way.^173,174^ Thereby, that gives powerful support for direct inhibition of cytokines for COVID-19 treatment.^175^ Anakinra is an IL-1 receptor antagonist (IL-1RA) to block the binding of IL-1 with their receptor, which has obtained FDA approval for the treatment of rheumatoid arthritis and neonatal onset multisystem inflammatory disease. Anakinra inhibited NLRP3 inflammasome-dependent pyroptosis, alleviated the lung pathology, and eventually reduced the mortality in the SASR-CoV-2-infected mice model.^176^ A variety of clinical studies evaluating the effects of anakinra for COVID-19 treatment have been conducted. In a retrospective study, compared to historical controls, anakinra reduced mechanical ventilation requirement and mortality (HR 0.22) in severe COVID-19.^177^ Furthermore, a non-randomized, single-arm phase II trial suggested that anakinra regulated the inflammatory system, attenuated the rate of ARDS (HR 0.3), and reduced 30-day mortality (HR 0.49) in COVID-19 (NCT4357366).^178^ Nevertheless, there is no significant difference between anakinra treatment and SoC for mild to moderate COVID-19 patients in a randomized controlled trial (NCT04341584).^179^ FDA-approved Canakinumab directly neutralizes IL-1β and has been investigated in clinical trials. Canakinumab showed positive effects on inflammatory markers, oxygen improvement, and final outcomes of COVID-19 patients according to the results of several retrospective studies.^180–183^ However, a randomized, double-blind, placebo-controlled phase III study indicated that canakinumab exhibited no benefit on mechanical ventilation requirement and mortality in hospitalized patients with severe COVID-19 (NCT04362813).^184^ In addition, Canakinumab has been evaluated in the COVID-19 patients concurrently with cardiac injury or type 2 diabetes, whose results have not been published (NCT04365153, NCT04510493).

Extensive studies have suggested that IL-6 greatly takes part in the progression of cytokine storm^185^ and the pathogenesis of COVID-19.^186^ The increased concentration of IL-6 has been identified as a negative prognosis factor for the severity and death rate of COVID-19 patients,^187,188^ giving support for the strategy of IL-6 blockage for COVID-19 treatment.^186^ Tocilizumab and sarilumab, FDA-approved humanized monoclonal antibodies, antagonize both soluble and membrane-bound IL-6 receptors to prevent IL-6 binding, in which the former has been utilized in cytokine release syndrome, giant cell arteritis, polyarticular juvenile idiopathic arthritis, rheumatoid arthritis, systemic sclerosis-associated interstitial lung disease. A large number of clinical trials of Tocilizumab and sarilumab have been conducted, whereas the efficacies are still controversial. According to the data of a randomized, SoC-controlled phase II trial, Tocilizumab utilized in COVID-19 pneumonia had no benefit on disease progression (NCT04346355).^189^ Concurrently, another randomized, double-blind, placebo-controlled trial also indicted that Tocilizumab was ineffective in reducing invasive mechanical ventilation and death of moderate COVID-19 patients (NCT04356937).^190^ On the contrary, a randomized, double-blind phase III trial displayed that lower percentage of mechanical ventilation requirement and 28-day mortality (HR 0.56) in Tocilizumab-treated COVID-19 pneumonia compared to placebo-controls (NCT04372186).^191^ Tocilizumab significantly had positive effects on the median number of organ support-free days (HR 1.64) and 90-day survival (HR 1.64) of severe COVID-19 with ICU support in a randomized, multifactorial adaptive platform trial (NCT02735707).^192^ Surprisingly, a large-scale trial gives the potent support for the reason that reduction in relative mortality (rate ratio (RR) 0.86), increase in discharge alive (RR 1.23), and decrease in invasive mechanical ventilation or death (RR 0.85) of Tocilizumab treated hospitalized COVID-19 compared to that in SoC-controls (NCT04381936).^193^ The chimeric monoclonal antibodies (such as Clazakizumab, Olokizumab, Siltuximab, and Sirukumab) inhibit IL-6 activity by reducing the binding of IL-6 with IL-6R. However, two randomized, double-blind, placebo-controlled phase II trials showed that Clazakizumab did not improve oxygen requirement and mortality of patients with severe SARS-CoV-2 infection (NCT04348500, NCT04343989). Equally disappointing results have emerged in the clinical trial of Olokizumab (NCT04380519). Meanwhile, a multicenter, randomized phase III trial indicated that there was no difference in time to clinical improvement (TTI) and mortality between Siltuximab-treated severe COVID-19 and controls (NCT04330638).^194^ Other phase II clinical trials of Clazakizumab (NCT04494724, NCT04363502) or Olokizumab (NCT05187793), Siltuximab (NCT04329650) for COVID-19 are under evaluation.

The loss of cellular ion homeostasis has been proven to promote NLRP3 inflammasome activation and cell pyroptosis during SARS-CoV-2 infection, thus ion-channel inhibitors (such as Amantadine, Memantine,^195^ Rimantadine,^196^ Tretinoin^197^) could be utilized in treatment with COVID-19 patients. Amantadine displayed antiviral activity via directly blocking the ion channel encoded by SARS-CoV-2 E protein in vitro.^198^ It is being investigated in a randomized, double-blind, controlled phase III trial for COVID-19 (NCT04894617).

Targeting overactivation of the Ang II-AT1R axis represents another strategy to attenuate NLRP3 inflammasome activation and cell pyroptosis in COVID-19. On one hand, ACE inhibitors (ACEIs, such as Lisinopril, Captopril) reduce the conversion of Ang I into Ang II, leading to a decrease in the level of Ang II. On the other hand, angiotensin receptor blockers (ARBs, such as Losartan, Irbesartan, Telmisartan, Olmesartan) can inhibit Ang binding with their receptors, leading to a reduction in the Ang-ATR signaling. Previous experiments^199–202^ and observational studies^203,204^ both provided the support that ACEIs and ARBs might be beneficial for COVI-19 patients. A randomized, SoC-controlled phase IV trial showed that telmisartan reduced mechanical ventilation requirement and death on days 15 and 30 in hospitalized patients with COVID-19 (NCT04355936).^205^ However, it is a pity that two randomized, double-blind, placebo-controlled phase II clinical trials of losartan had no significant effect on outpatients and mild hospitalized patients with COVID-19 (NCT04311177, NCT04340557).^206,207^ Therefore, more clinical trials to exactly evaluate the effects of ACEIs and ARB on COVID-19 treatment are ongoing (NCT04345406, NCT04591210, NCT02735707, NCT04920838, NCT04466241).

## NETosis and SARS-CoV-2

### NETosis in SARS-CoV-2 infection

NETosis is a ROS-dependent PCD driven by neutrophil extracellular traps (NETs) in response to various infections, which was first described by A. Zychlinsky in 2004.^208^ Peptidylarginine deiminase 4 (PAD4), neutrophil elastase (NE), GSDMD, and free DNA greatly participate in the whole process of NETosis.^209^ NETs are complex networks comprised of DNA containing histones, myeloperoxidase (MPO), and NE.^210^

**Fig. 4:**
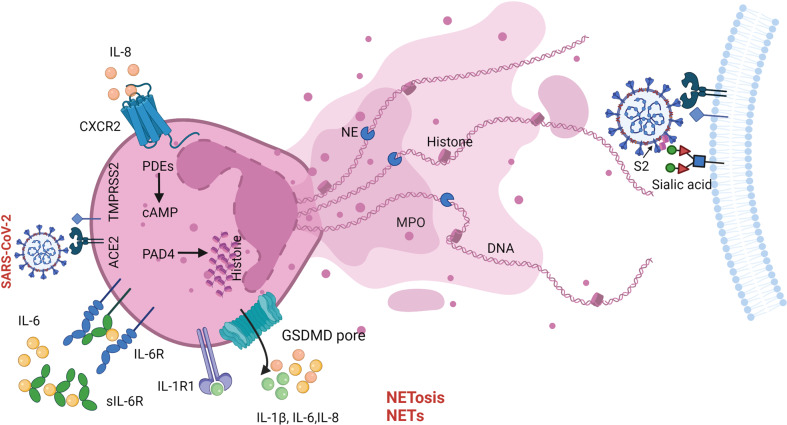
The NETosis during SARS-CoV-2 infection. NETs, released by SARS-CoV-2-induced NETosis, are comprised of DNA, histones, MPO, and NE. The IL-8-CXCR2, IL-6-IL-6R, and IL-1β-IL-1R1 axis play important roles in recruiting and activating neutrophils. In addition, SARS-CoV-2 hijacks histones to interact with S2 and sialic acid for enhanced infection. SARS-CoV-2 severe acute respiratory syndrome coronavirus 2, S2 subunit 2 of SARS-CoV-2 spike protein, ACE2 angiotensin-converting enzyme-2, TMPRSS2 transmembrane serine protease 2, IL-8 interleukin-8, CXCR2 C-X-C motif chemokine receptor 2, sIl-6R soluble IL-6 receptor, IL-1R1 interleukin-1 receptor 1, GSDMD gasdermin-D, PDEs phosphodiesterases, cAMP cyclic adenosine monophosphate, PAD4 peptidylarginine deiminase 4, NE neutrophil elastase, MPO myeloperoxidase, NETs neutrophil extracellular traps. Created with BioRender

Cytokine storm has been characterized in COVID-19, particularly in severe patients. Previous studies have suggested that the pro-inflammatory cytokines (such as IL-1β, IL-6, C-X-C motif chemokine 8 (CXCL-8)/IL-8) can potently recruit and activate neutrophils.^211,212^ Accumulative studies have shown a significant increase in the numbers and activation of neutrophils, which are associated with severity during SARS-CoV-2 infection.^213,214^ The increased neutrophils and NETosis were found in blood samples from COVID-19 patients via transcriptomics, proteomics, and cfDNA analyses.^215^ In addition, the autocrine loop of IL-8 is identified in severe COVID-19 patients for augmentation of neutrophil migration and activation.^216^ Meanwhile, the concentrations of NETs in plasma, tracheal aspirate, and lung specimens from COVID-19 patients were higher compared to the healthy controls.^217^ SARS-CoV-2 can directly stimulate NETosis of healthy neutrophils, which is dependent on ACE2, TMPRSS2, and PAD4. As a result, the accumulation of NETs leads to apoptosis in lung epithelial cells. Furthermore, a recent study has indicated that SARS-CoV-2 cunningly manipulate histones (especially H3 and H4) released by NETosis to bridge subunit 2 of the S protein and sialic acid on the cell surface and promote membrane fusion, finally boosting their infectivity.^218^ Taken together, NETosis have two-edged sword activities during virus infection.^219,220^ The NETs trap viral particles to inhibit their transmission efficiently. However, the NETs also lead to cell damage, cytokine storm,^221^ and even immunothrombosis.^222^ The immunothrombosis is comprised of activated leukocytes with platelets and plasma coagulation factors and is involved in systemic diseases of COVID-19.^126,223,224^ The NETosis occurred during SARS-CoV-2 infection is displayed in Fig. 4.

### NETosis-targeted therapies

Thereby, it has been proposed that inhibiting neutrophils and NETosis might be feasible approaches for COVID-19 treatment.^225^ Certainly, the primary strategy focuses on the vital molecules involved in NETosis, such as PAD4, NE, and GSDMD. The PAD4 inhibitors (such as Cl-amidine and GSK484) prevent NETosis by reducing histone citrullination in the treatment of severe inflammatory diseases.^226^ Sivelestat, a potent NE inhibitor approved in Korea and Japan, can ameliorate acute lung injury.^227^ A retrospective cohort study showed that sivelestat exhibited positive outcomes in patients with ARDS,^228^ indicating that NE inhibitor might be beneficial for patients with COVID-19. In addition, A randomized, placebo-controlled phase Ib/II clinical trial of Alvelestat treatment for COVID-19 with ARDS has been completed with no results published (NCT04539795). The degradation and elimination of NETs using DNases represent an appropriate pathway to reduce NETs’ burden. Dornase alfa is a FAD-approved recombinant human DNase I and showed significant effects of antiviral activity and disease relief of SARS-CoV-2 infection in vitro and in vivo.^229^ Currently, there are several clinical trials related dornase alfa for COVID-19 (NCT04432987, NCT04409925, NCT04409925, NCT04445285, NCT04402970, etc). In a non-randomized phase III clinical trial (NCT04402970), inhaled dornase alfa yielded positive outcomes of improved oxygenation and decreased NETs in COVID-19 patients.^230^

In addition to therapies directly antagonizing the forming and degradation, it is feasible to hinder the components involved in amplification loops of NETs. It is acknowledged that IL-8-C-X-C motif chemokine receptor 2 (CXCR2) axis powerfully recruits neutrophils and promotes NETs formation.^231^ However, the clinical trial of anti-IL-8 monoclonal antibody BMS-986253 for COVID-19 has been terminated according to an interim analysis that suggested its ineffectiveness (NCT04347226). Furthermore, there are no registered trials of CXCR2 inhibitors for the treatment of COVID-19 patients.

Phosphodiesterases (PDEs) regulate the levels of cyclic adenosine monophosphate (cAMP) and cyclic guanosine monophosphate (cGMP) and play a vital role in inflammation.^232^ The cAMP-specific PDE4 is highly expressed and promotes inflammation in neutrophils. Thus, several PDE inhibitors are considered as possible drugs for COVID-19 treatment.^233^ The phase III clinical trials of PDE4 selective-inhibitor apremilast and ensifentrine (NCT04590586, NCT02735707) for COVID-19 treatment are ongoing. Dipyridamole and Pentoxifylline are FDA-approved nonspecific PDE inhibitors. It is reported that dipyridamole suppresses SARS-CoV-2 replication in infected cells and contributes to disease relief with significantly reduced D-dimer in COVID-19 patients.^234^ The randomized phase II (NCT04391179) and III (NCT04410328) clinical trials to evaluate the outcomes of dipyridamole for COVID-19 patients have been completed without results published. The decreased serum lactate dehydrogenase (LDH) level and increased lymphocyte count were observed in COVID-19 patients receiving pentoxifylline treatment.^235^ The efficacy of pentoxifylline for COVID-19 is being evaluated in the clinical trial (NCT04433988).

## Ferroptosis and SARS-CoV-2

### Ferroptosis in SARS-CoV-2 infection

Ferroptosis is a new-type PCD mainly caused by ROS-dependent lipid peroxidation, which was first systematically described by R. Stockwell in 2012.^236^ The morphology of ferroptosis is indicated by smaller mitochondria with increased membrane densities, reduced crista, and ruptured outer membrane. Ferroptosis has been implicated in numerous organ injuries and degenerative pathologies.^237^ Ferroptosis can be triggered through two main pathways: the extrinsic pathway (also known as the transporter-dependent pathway) and the intrinsic pathway (also known as the enzyme-regulated pathway). Iron uptake is initiated by transferrin receptor 1 (TfR1)-dependent endocytosis and Fe^2+^ is released into cytoplasm via six-transmembrane epithelial antigen of prostate 3 (Steap3) and divalent metal transporter 1 (DMT1). Intracellular iron is stored in ferritin (which can further release Fe^2+^ via ferritinophagy) and exported to the extracellular by ferroportin. Meanwhile, iron overload leads to mitochondrial ROS generation. Polyunsaturated fatty acids (PUFAs) are synthesized to phospholipid hydroperoxides (PLOOHs) mediated by acyl-CoA synthetase long-chain family member 4 (ACSL4), lysophosphatidylcholine acyltransferase 3 (LPCAT3), and lipoxygenases (LOXs), termed as lipid peroxidation, resulting in ferroptosis. The cellular antioxidant system exhibits ferroptosis-suppressing effects, particularly the cystine-glutathione (GSH)-glutathione peroxidase 4 (GPX4) axis with potent inhibition of lipid peroxidation.

**Fig. 5:**
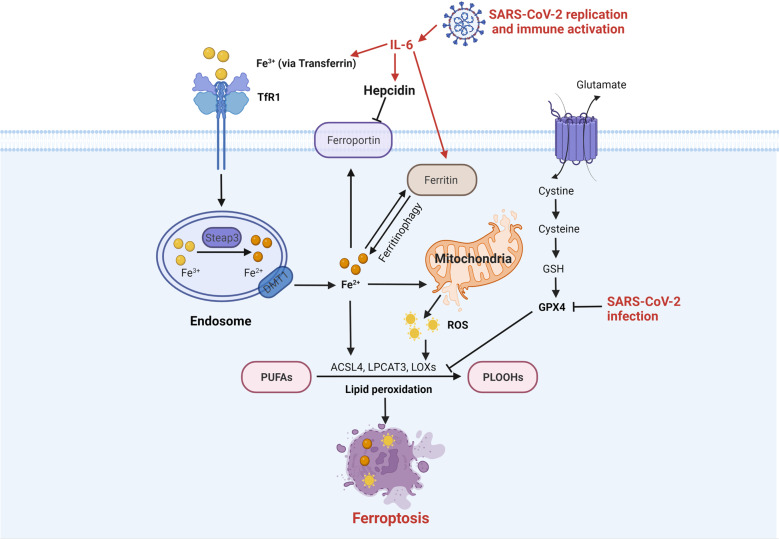
The ferroptosis upon SARS-CoV-2 infection. Ferroptosis is triggered by lipid peroxidation resulting from the imbalance among iron metabolism, ROS generation, and antioxidant system (especially the cystine-GSH-GPX4 axis). The high level of IL-6 in COVID-19 directly promotes the expression of iron accumulation proteins (including transferrin and ferritin) and indirectly inhibits the exported protein ferroportin via hepcidin, leading to cell ferroptosis. Meanwhile, SARS-CoV-2 infection exhibits a negative effect on GPX4. SARS-CoV-2 severe acute respiratory syndrome coronavirus 2, IL-6 interleukin-6, TfR1 transferrin receptor 1, Steap3 six-transmembrane epithelial antigen of prostate 3, DMT1 divalent metal transporter 1, ROS reactive oxygen species, PUFAs polyunsaturated fatty acids, PLOOHs phospholipid hydroperoxides, ACSL4 acyl-CoA synthetase long-chain family member 4, LPCAT3 lysophosphatidylcholine acyltransferase 3, LOXs lipoxygenases, GSH glutathione, GPX4 glutathione peroxidase 4. Created with BioRender

Mounting evidence has suggested that ferroptosis takes part in the pathogenesis of COVID-19. Ferroptosis was detected in samples of human hearts^238^ and hamsters’ lungs^239,240^ with SARS-CoV-2 infection. The changes of iron metabolism markers in blood samples (including decreased serum iron, and increased ferritin) demonstrated iron overload and are associated with severe COVID-19.^241–244^ The secretion of IL-6 is highly induced by inflammation upon SARS-COV-2 infection. Previous studies have shown the effects of IL-6 on iron metabolism. On the one hand, IL-6 can directly boost transferrin uptake and ferritin expression^245^; on the other hand, IL-6 can induce the synthesis of hepcidin,^246^ an inhibitor of ferroportin, consequently leading to cellular iron accumulation. Several studies showed that increased serum hepcidin correlated with the severity of COVID-19.^244,247^ In addition, the scRNA-seq data of PBMC, T cells, and B cells from COVID-19 patients revealed that ferroptosis-related genes (including GPX4, FTH1, FTL, and SAT1) were increased in the acute phase and decreased in the recovery phase.^55^ Besides, SARS-CoV-2 was reported to downregulate the GPX4 mRNA level in infected Vero E6 cells.^248^ The induction of ferroptosis by SARS-CoV-2 could be reversed through two ACSL4 inhibitors in vitro.^249^ The ferroptosis triggered by SARS-CoV-2 is shown in Fig. 5.

### Ferroptosis-targeted therapies

Given the harmfulness of ferroptosis in COVID-19, it is reasonable to protect cells from ferroptosis via three aspects: alleviate iron overload^250^; augment the cystine-GSH-GPX4 axis; inhibit lipid peroxidation. Deferoxamine, an FDA-approved injective iron chelator, binds with free iron and iron from lysosomal ferritin, forming a stable complex removed by kidneys.^251,252^ Deferiprone is another iron chelator approved by FAD for use of oral, being more powerful for the chelation of iron in the heart compared to deferoxamine. The clinical trials of iron chelation therapy for COVID-19 are ongoing (NCT04333550, NCT04361032, NCT04389801). Intriguingly, the available data suggested that the selenium level is positively associated with clinical complications and outcomes,^253,254^ which supports selenium supplementation in COVID-19 patients.^255^ Selenium was reported to induce GPX4 expression to protect cells from ferroptosis in the model of stroke^256^ and further applied for COVID-19 (NCT04869579, NCT04798677, NCT04751669, NCT04323228). Ebselen, a synthetic selenium compound, can not only impede ferroptosis by acting as a GPX mimic but also inhibit virus replication by interacting with SARS-CoV-2 M^pro^.^257,258^ N-acetylcysteine (NAC), a precursor of GSH, possesses anti-ferroptosis activity through directly reinforcing the cystine-GSH-GPX4 axis^259^ and reducing the IL-6-promoted ROS production,^260^ accounting for a part of its effects for COVID-19 treatment. Additionally, it has been proven that NAC can regulate T cell response^261^ and inflammation.^261^ However, in a randomized, double-blind, placebo-controlled trial, high-dose NAC failed to prevent the progression of ARDS in COVID-19 patients.^262^ More clinical trials of NAC as monotherapy and combination therapy for COVID-19 are still ongoing (NCT04374461, NCT04928495, NCT04792021, NCT04703036, NCT05074121, NCT04455243).

It is well known that lipid peroxidation is the most important step in ferroptosis, thus blocking key enzymes (such as ACSL4 and LOX^263^) of this process as a potential approach for COVID-19 treatment. Pioglitazone and Troglitazone, two FDA-approved thiazolidinediones for diabetes, can reduce cell ferroptosis and viral replication by selectively inhibiting ACSL4 activity during SARS-CoV-2 infection.^264,265^ Two randomized phase IV clinical trials of pioglitazone utilized in patients with COVID-19 concurrently with type 2 diabetes are recruiting (NCT04604223 and NCT04535700).

## Autophagy and SARS-CoV-2

### Autophagy core machinery

Autophagy (also named type II PCD) is a highly conserved catabolic process to degrade and recycle misfolded proteins and damaged organelles to maintain cell homeostasis and eliminate intracellular pathogens, which was first elucidated in yeast by Ohsumi 1992.^266,267^ The morphological characteristics of autophagy manifest cytoplasmic vacuoles and autophagic bodies. Autophagy is classified into three types: macroautophagy (referred to as autophagy), microautophagy, and selective autophagy. In canonical autophagy, subcellular membranes (such as ER, Golgi complex, mitochondria, and endosomes) engulf the undesired components to form a double-membrane structure named phagophore. The phagophore membrane expands and elongates until it closes in on itself to form the autophagosome, which further fuse with lysosome to form the autophagolysosome for degradation under acidic conditions.^268^ The whole process is regulated by a series of proteins encoded by autophagy‐related genes (ATGs),^269^ mainly including: (i) the unc-51 like autophagy activating kinase 1 (ULK1) complex; (ii) the phosphatidylinositol 3-phosphate class III lipid kinase (PIK3C3) complex; (iii) the ubiquitin-like ATG12-conjunction system; (iv) the ubiquitin-like microtubule-associated protein 1 light chain 3 (MAP1LC3, termed as LC3)-conjunction system. Besides, the undesired components are labeled by sequestosome 1 (SQSTM1, also named as p62) complex and brought into the autophagosome for degradation. In addition, the fusion step is mediated by the homotypic fusion and protein sorting (HOPS) complex and N-ethylmaleimide sensitive factor attachment protein receptor (SNARE), and vesicle associated membrane protein 8 (VAMP8) complex.

### Autophagy induction by SARS-CoV-2

Growing studies have suggested that the interplay of SARS-CoV-2 and autophagy is extremely intricate, which has not been fully established. The double-membrane vesicles (DMVs) were detected by transmission electron microscopy^270^ and further confirmed via integrative imaging (including FIB-SEM analysis, electron tomography, and 3D rendering)^271^ in SARS-CoV-2-infected nasal, bronchial, and pulmonary human epithelial cells. SARS-CoV-2 NSP6, which co-localizes in ER, has been reported to promote rearrangements of ER membranes to DMVs production.^272^ Thus, like other coronaviruses,^273^ SARS-CoV-2 can hijack autophagy flux to form DMVs derivated from ER membranes. As of current studies, these DMVs provide the niches for viral RNA replication and immune escape. In addition, recent evidence has concluded that viral proteins encoded by SARS-CoV-2 can promote autophagy to regulate the host’s immune response. It has been reported that SARS-CoV-2 M protein translocates to mitochondria via mitochondrial Tu translation elongation factor (TUFM) and interacts with LC3-II through its LC3-interacting region (LIR), thus inducing mitochondrial autophagy (termed as mitophagy).^274^ In a similar way, SARS-CoV-2 ORF10 directly interacts with a mitochondrial receptor Bcl-2 interacting protein 3 like (BNIP3L)/NIX (also containing LIR) to promote mitophagy.^275^ On the one hand, activated mitophagy causes the degradation of mitochondrial antiviral signaling protein (MAVS). Previous studies have indicated that MAVS can activate transcription factors interferon regulatory factor 3 (IRF3) and NF-κB to upregulate the expression of immune response and inflammation-related genes.^276^ Thus, SARS-CoV-2 M and ORF10 abate type I IFN response and pro-inflammatory cytokines by inducing mitophagy-mediated MAVS degradation. On the other hand, mitophagy decreases the release of mtDNA and ROS, thus inhibiting NLRP3 inflammasome-mediated cell pyroptosis and cytokine secretion. Additionally, type I IFN production is also diminished due to SARS-CoV-2 NSP13 recruiting TBK1 (via RecA domain) to p62 (via ZBD domain) for autophagic degradation of TBK1. Interestingly, the direct interaction of SARS-CoV-2 ORF8 with major histocompatibility complex I (MHC I) leads to MHC I lysosomal degradation, reduction of antigen presentation, and evading immune surveillance in SARS-CoV-2-infected cells and mice model.^277^

### Autophagy inhibition by SARS-CoV-2

**Fig. 6:**
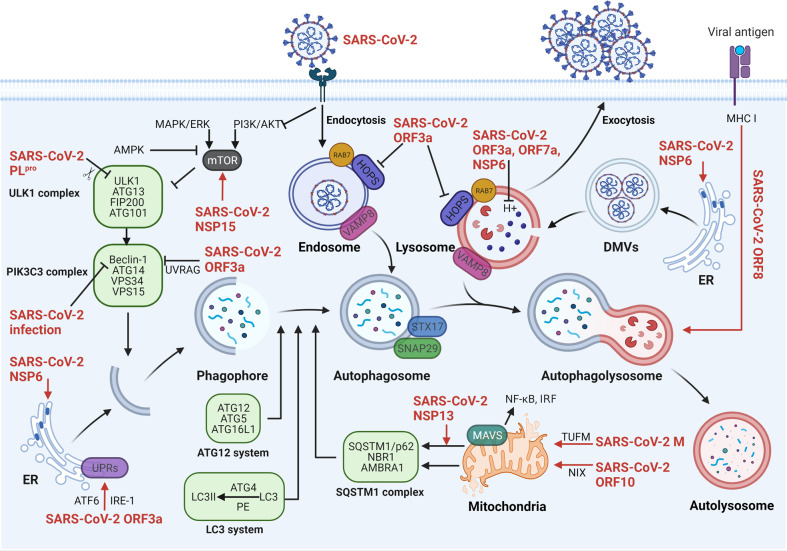
The complicated mechanisms of SARS-CoV-2 manipulating cell autophagy. The phagophore derived from subcellular membranes expands and elongates to form the autophagosome, further fusing with lysosome to form the autophagolysosome for degradation, whose processes are closely regulated by ATGs. SARS-CoV-2 hijacks ER-derivated DMVs for replication and immune escape, which are enhanced by SARS-CoV-2 NSP6. SARS-CoV-2 M protein (via TUFM), ORF10 (via NIX), and NSP13 can induce mitochondrial autophagy to reduce the activation of NF-κB and IRF. Besides, SARS-CoV-2 ORF8 promotes MHC I degradation. In contrast, SARS-CoV-2 also plays an important role in autophagy inhibition. SARS-CoV-2 PL^pro^-cleaved ULK1 and SARS-CoV-2 NSP15-activated mTOR cause ULK1 complex inhibition. SARS-CoV-2 ORF3a and SARS-CoV-2 infection both regulate the PIK3C3 complex, all of which lead to autophagy inhibition. SARS-CoV-2 ORF3a, ORF7a, and NSP6 impede the fusion of lysosome with autophagosome via damaging lysosomal acidification. Besides, SARS-CoV-2 ORF3a directly impairs the interactions among membrane fusion complex HOPS, SNARE and VAMP8. SARS-CoV-2 severe acute respiratory syndrome coronavirus 2, M membrane, ORF open reading frame, NSP nonstructural protein, PL^pro^ papain-like protease, ULK1 unc-51 like autophagy activating kinase 1, ATG autophagy‐related gene, FIP200 focal adhesion kinase family interacting protein of 200 kD, PIK3C3 complex phosphatidylinositol 3-phosphate class III lipid kinase complex, UPRs unfolded protein responses, ATF6 activating transcription factor 6, IRE-1 inositol-requiring enzyme 1, MAPK mitogen-activated protein kinase, ERK extracellular-signal regulated kinase, PI3K phosphoinositide 3-kinase, AKT protein kinase B, mTOR mammalian target of rapamycin, UVRAG UV irradiation resistance-associated gene, LC3 microtubule-associated protein 1 light chain 3, PE phosphatidylethanolamine, SQSTM1 sequestosome 1, NBR1 neighbor of BRCA1 gene 1, Ambra1 Beclin-1-regulated autophagy, HOPS homotypic fusion and protein sorting, RAB7 Ras-related protein Rab7, STX17 syntaxin 17, SNAP29 synaptosome associated protein 29, VAMP8 vesicle associated membrane protein 8, MAVS mitochondrial anti-viral signaling protein, NF-κB nuclear factor kappa B, IRF transcription factors interferon regulatory factor, TUFM mitochondrial Tu translation elongation factor, NIX Bcl-2 interacting protein 3 like, DMVs double-membrane vesicles, MHC I major histocompatibility complex I, ER endoplasmic reticulum. Created with BioRender

A great number of reports have demonstrated that autophagy is an anti-viral process, for reason that it not only impedes the viral life cycle by eliminating viral particles and viral components (also known as virophage) but also mediates antigen presentation to induce adaptive immunity. Therefore, SARS-CoV-2 could inhibit autophagy through multifarious mechanisms to facilitate its replication and transmission.^278^ SARS-CoV-2 ORF3a prohibits cell autophagy in a variety of ways. SAS-CoV-2 ORF3a interacting with autophagy regulator UV irradiation resistance-associated gene (UVRAG)^279^ promotes PI3KC3-C1 but blocks PI3KC3-C2, leading to incomplete autophagy.^280^ Incomplete autophagy represents the inhibition of autophagosome-lysosome fusion, that is autophagosome induction but autolysosome reduction, also termed as the failure of autophagosome turnover. SAS-CoV-2 ORF3a-induced ER stress can also trigger incomplete autophagy by activating transcription factor 6 (ATF6)- and inositol-requiring enzyme 1 (IRE-1)-dependent unfolded protein response (UPR).^281^ Moreover, another two studies have demonstrated that SARS-CoV-2 ORF3a directly interacts with and sequestrates VPS39 in endosomes and lysosomes, resulting in the dysfunctional HOPS complex. For one thing, VPS39 sequestration disturbs the interaction of HOPS with Ras-related protein Rab-7 (RAB7).^282^ For another, invalid HOPS blocks the assembly of SNARE complex via directly interacting with STX17,^283^ thereby preventing the fusion of autophagosomes with lysosomes. In addition, SARS-CoV-2 ORF3a can promote viral egress through lysosomal exocytosis.^114^ This process is regulated by Ca^2+^ channel TRPML3, lysosomal trafficker BORC-ARL8b complex, and exocytosis-related STX4-SNAP23-VAMP7 SNARE complex. Like ORF3a, SARS-CoV-2 ORF7a also co-localizes with late endosomes and disturbs lysosomal acidification, resulting in blockage of autophagosome turnover.^284^ Besides, SARS-CoV-2 NSP15 was reported to affect the production of autophagosomes via interacting with the mTOR axis, which could be reversed by rapamycin (an mTOR inhibitor).^284^ It is known that the mTOR axis exerts inhibitory effects on autophagy via prohibiting the ULK1 complex.^285^ Meanwhile, SARS-CoV-2 PL^pro^ protein directly cleaves ULK1 to the destruction of ULK1-ATG13 complex formation.^286^ The complicated mechanisms of cell autophagy mediated by SARS-CoV-2 are summarized in Fig. 6.

Taken together, cell autophagy has a double face during SARS-CoV-2 infection. On the one hand, autophagy exerts its anti-viral activity by induction of virophage and regulation of immune response; On the other hand, autophagy is manipulated by SARS-CoV-2 to favor their replication and transmission, immunosurveillance escape, and pro-inflammatory response. Furthermore, accumulative autophagosomes would induce the Bcl-2/BAX-mediated cell apoptosis and the NLRP3 inflammasome-dependent cell pyroptosis (see previous sections), resulting in cell damage and tissue injury.

### Autophagy-targeted therapies

Although the intricate relation is still obscure, cell autophagy is important for SARS-CoV-2 infection and host response, providing rationality for autophagy mediators as a therapeutic strategy. There are a large number of drugs under clinical investigation. It should be noted that most of them exert anti-viral activity by regulating autophagy as well as other mechanisms. Chloroquine (CQ) and its less toxic derivative, hydroxychloroquine (HCQ), two FDA-approved anti-malarial drugs, exhibit autophagy inhibition, immunosuppressive functions, anti-inflammatory properties as well as anti-viral activity, which has been proposed against SARS-CoV,^287^ HIV,^288^ and Zika.^289^ The previous study has shown that they can inhibit autophagic flux by disturbing autophagosome-lysosome fusion^290^ and endosomal/lysosomal acidification,^291^ resulting in accumulative autophagosomes-induced cell death and cessation of virus replication. In addition, they could impede SARS-CoV-2 entry by restricting the glycosylation of ACE2 and S protein^292^ and inhibiting acidification-dependent endocytosis.^293^ Several in vitro studies showed that CQ and HCQ made inhibitory effects on SARS-CoV-2-infected Vero E6 cells.^293–295^ A prospective observational study indicated that CQ provided positive effects on reduced viral load and time to clinical recovery (TTCR) in COVID-19 patients, compared with historical controls.^296^ A randomized clinical trial showed that HCQ treatment improved the TTCR and absorption of pneumonia in COVID-19 patients, compared with SoC-treated patients.^297^ In addition, another non-randomized, small-size trial suggested that HCQ decreased viral load in COVID-19, whose effect could be reinforced by azithromycin.^298^ These positive results pay the way to further investigation of CQ and HCQ in large-scale and randomized clinical trials. Nevertheless, the majority of them presented disappointing outcomes. In a randomized, SoC-controlled phase II/III clinical trial, HCQ exhibited no significant effect on the 28-day mortality and hospital stay duration in hospitalized patients with COVID-19 (NCT04381936).^299^ Another randomized, double-blind phase III trial also obtained negative results that no significant difference at day 14 in hospitalized COVID-19 treated with HCQ and placebo (NCT04332991).^300^ In light of current results, it is indicated that the anti-viral effects of CQ/HCQ are controversial. Given its diverse effects, CQ/HCQ are still considered as potential drugs, thus employment of combination therapies and pre/post-exposure prophylaxis might be the further direction. Furthermore, autophagy inhibition can be achieved by inhibiting autophagy-related complexes, among which targeting the ULK1 complex and PIK3C3 complex are most studied. Consequently, several studies showed that PIK3C3 complex inhibitors potently reduced SARS-CoV-2 replication,^301,302^ among which commonly applied to impede autophagy are VPS34-IN1, VVPS34-IN1, 3-methyladenine,^303^ wortmannin, LY294002,^304^ PT210,^305^ and GSK-2126458,^306^. Inhibitors of the ULK1 complex (including ULK-101,^307^ compound 6,^308^ MRT68921,^309^ and SBI-0206965^310^) are also identified as autophagy inhibitors and need further investigation for antagonizing SARS-CoV-2 infection.

In the light of uncontrolled inflammatory response in severe COVID-19, the administration of autophagy activators for anti-inflammation and anti-virus also might be helpful. It is known that mTOR signaling plays a vital role in mediating cell autophagy. AKT and MAPK-activated mTOR can inhibit autophagy, whereas AMPK and p53-suppressed mTOR induce autophagy. The proteotranscriptomic data displayed activation of the PI3K/AKT/mTOR pathway in response to SARS-CoV-2 infection.^311^ In line with this result, the mTOR activation was confirmed by in-depth analyses, and AKT inhibitor MK-2206 inhibited SARS-CoV-2 replication.^312^ Rapamycin (also known as Sirolimus), an FDA-approved mTOR inhibitor, showed positive anti-viral activity on porcine epidemic diarrhea virus,^313^ transmissible gastroenteritis virus,^314^ H1N1,^315^ and MERS-CoV^316^ in previous studies. The clinical trials investing the effects of sirolimus treatment for COVID-19 are ongoing (NCT04461340, NCT04341675, NCT04482712). Metformin, an FDA-approved antidiabetic drug, promotes autophagy through upregulation of AMPK and downregulation of mTOR.^317,318^ Furthermore, increasing evidence has demonstrated that it can directly block viral entry and infection, and mediate inflammatory and immune responses,^319,320^ which provides a basis for rational application in COVID-19 patients. In a retrospective analysis, 30-day mortality was significantly decreased (HR 0.48) in nursing home patients with metformin treatment, compared to that in patients with no metformin-containing regimens.^321^ Other retrospective studies showed similar results in hospitalized patients with SARS-CoV-2 infection.^322,323^ A randomized, double-blind, placebo-controlled phase II trial of metformin for hospitalized COVID-19 patients with ARDS has been completed with no result published yet (NCT04625985). Other clinical trials of metformin-treated with COVID-19 are ongoing (NCT04604678, NCT04510194, NCT04727424). Additionally, a number of drugs (such as resveratrol, trehalose, ivermectin, nitazoxanide, etc) with the ability to induce autophagy to anti-virus have been under investigation. However, the underlying mechanisms of these drugs for COVID-19 treatment still have been obscure.

## Conclusions and perspectives

Taken together, SARS-CoV-2 can regulate cell death and cell autophagy through various mechanisms and participate in the occurrence and development of COVID-19. From the above description, it is not hard to conclude that cell death and autophagy are a double-edged sword. On the one hand, they can directly prevent the propagation of viruses through the reduction in replicative vehicles. On the other hand, they would lead to tissue destruction, widespread virus, cytokine storm, and incompetent immune response. At different times of infection, SARS-CoV-2 can craftily manipulate cell death and autophagy to serve itself. Thus, it should be carefully decided whether and when cell death and autophagy should be inhibited or stimulated. With the help of massive inhibitors and agonists targeting these pathways, further researches are able to dive into the diverse influences of cell death and autophagy on COVID-19 development. In addition, high-throughput methods including single-cell sequencing, proteomics and metabonomics, and spatial omics may facilitate the discovery of novel pathways in COVID-19 and the cross-talk among different cell death and autophagy pathways. A good question is whether disparate cell death and autophagy pathways are induced in different strains of SARS-CoV-2. More delicate observations are needed to figure out the relationship between SARS-CoV-2 mutations and altered cell death and autophagy pathways.

In view of the important roles of cell death and autophagy, a variety of related therapeutic strategies have been explored and put into practice in clinical trials, whereas the results are controversial (Table 1). Thereby, the interactions among SARS-CoV-2 infection, host response, and therapies need more in-depth and comprehensive research. Meanwhile, it is indeed crucial to estimate the therapeutic window for COVID-19 patients according to the course and severity of the disease. The real-time and dynamic information on clinical manifestations, laboratory parameters, and imaging findings, combined with relevant high-throughput methods may be helpful in finding the critical point for therapies. It may be hard but beneficial to create a guide to balance ameliorating symptoms and preventing viruses from spreading in patients.

**Table 1:** Therapeutic targets and drugs related to cell death and their clinical trials for COVID-19.

Target	Drug	Effect on cell death	Clinical trial ID	Clinical trial result
c-FLIP	LBH589 (histone deacetylase inhibitor)	(+) Apoptosis	/	/
NF-κB	Phillyrin, Dexamethasone, Macrolide antibiotics (antioxidative agent)	(+) Apoptosis; (−) Pyroptosis	/	/
Caspase	Emricasan	(−) Apoptosis	NCT04803227	Terminated
TNF-α	Infliximab	(−) Apoptosis, Necroptosis, Pyroptosis	NCT04425538	(−) Inflammatory cytokines
			NCT04922827, NCT05220280, NCT04593940, NCT05273242	Ongoing
	Adalimumab		IRCT20151227025726N23	Negative
	Etanercept		NCT05080218	Ongoing
RIPK1	Primidone	(−) Necroptosis	/	/
RIPK3	GSK872	(−) Necroptosis	/	/
MLKL	Necrosulfonamide	(−) Necroptosis	/	/
NLRP3	MCC950	(−) Pyroptosis	/	/
	DFV890		NCT04382053	Negative
	Dapansutrile		NCT04540120	Ongoing
	Colchicine (microtubule polymerization inhibitor)		RBR-8jyhxh	(−) Oxygenation and hospitalization
			NCT04322682	(−) Hospitalization and death
			NCT04381936	Negative
			NCT04328480	Negative
GSDMD	Disulfiram (other targets: M^pro^, PL^pro^, NSP13, and NSP14)	(−) Pyroptosis	NCT04594343	ND
			NCT04485130	Ongoing
	Dimethyl fumarate (another target: NRF2)		NCT04381936, NCT04792567	Ongoing
IL-1β-IL-1β receptor	Anakinra	(−) Pyroptosis, NETosis	Retrospective study	(−) Mechanical ventilation requirement and mortality
			NCT4357366	(−) ARDS and 30-day mortality
			NCT04341584	Negative
	Canakinumab		Retrospective study	(−) Inflammatory markers, (+) oxygenation and final outcomes
			NCT04362813	Negative
			NCT04365153	ND
			NCT04510493	ND
IL-6- IL-6 receptor	Tocilizumab	(−) Pyroptosis, NETosis	NCT04346355	Negative
			NCT04356937	Negative
			NCT04372186	(−) Mechanical ventilation requirement and 28-day mortality
			NCT02735707	(+) Median number of organ support-free days and 90-day survival
			NCT04381936	(−) Relative mortality, (+) discharge alive, (−) invasive mechanical ventilation or death
	Sarilumab		/	/
	Clazakizumab		NCT04348500	Negative
			NCT04343989	Negative
			NCT04494724, NCT04363502	Ongoing
	Olokizumab		NCT04380519	Negative
			NCT05187793	Ongoing
	Siltuximab		NCT04330638	Negative
			NCT04329650	Ongoing
	Sirukumab		/	/
Ion channel	Amantadine	(−) Pyroptosis, Apoptosis	NCT04894617	Ongoing
	Memantine, Rimantadine, Tretinoin		/	/
Ang II-AT1R receptor	Lisinopril, Captopril (ACEI)	(−) Pyroptosis	NCT04345406, NCT04591210, NCT02735707	Ongoing
	Telmisartan (ARB)		NCT04355936	(−) Mechanical ventilation requirement and 15, 30-day death
			NCT04920838, NCT04466241	Ongoing
	Losartan (ARB)		NCT04311177	Negative
			NCT04340557	Negative
	Irbesartan, Olmesartan (ARB)		/	/
PAD4	Cl-amidine, GSK484	(−) NETosis	/	/
Neutrophil elastase	Sivelestat	(−) NETosis	Retrospective study	(+) Oxygenation
	Alvelestat		NCT04539795	ND
DNA	Dornase alfa	(−) NETosis	NCT04402970	(+) Oxygenation
			NCT04432987, NCT04409925, NCT04445285,	Ongoing
IL-8-CXCR2	BMS-986253	(−) NETosis	NCT04347226	Terminated
PDE	Apremilast, Ensifentrine (PDE4 inhibitor)	(−) NETosis	NCT04590586, NCT02735707	Ongoing
	Dipyridamole (pan PDE inhibitor)		ChiCTR2000030055	(−) D-dimer and viral load, (+) disease relief
			NCT04391179	ND
			NCT04410328	ND
	Pentoxifylline (pan PDE inhibitor)		/	(−) LDH, (+) number of lymphocyte
			NCT04433988	Ongoing
Iron	Deferoxamine, Deferiprone	(−) Ferroptosis	NCT04333550, NCT04361032, NCT04389801	Ongoing
GPX4	Selenium	(−) Ferroptosis	NCT04869579, NCT04798677, NCT04751669, NCT04323228	Ongoing
	Ebselen (another target: M^pro^)		/	/
GSH	N-acetylcysteine (antioxidative and anti-inflammatory agent; T cell response regulator)	(−) Ferroptosis, Pyroptosis, NETosis	U1111-1250-356	Negative
			NCT04374461, NCT04928495, NCT04792021, NCT04703036, NCT05074121, NCT04455243	Ongoing
ACSL4	Pioglitazone, Troglitazone (antidiabetic agent)	(−) Ferroptosis	NCT04604223, NCT04535700	Ongoing
Autophagosome-lysosome fusion; endosomal/lysosomal acidification	Chloroquine (anti-malarial agent; immunosuppressive and anti-inflammatory agent)	(−) Autophagy, Pyroptosis, NETosis	Prospective observational study	(−) Viral load and time to clinical recovery
	Hydroxychloroquine		ChiCTR2000029559	(−) Time to clinical recovery
			2020-000890-25	(−) Viral load
			NCT04381936	Negative
			NCT04332991	Negative
ULK1 complex	ULK-101, Compound 6, MRT68921, SBI-0206965	(−) Autophagy	/	/
PIK3C3 complex	VPS34-IN1, VVPS34-IN1, 3-methyladenine, Wortmannin, LY294002, PT210, GSK-2126458	(−) Autophagy	/	/
mTOR	MK-2206 (AKT inhibitor)	(+) Autophagy	/	/
	Rapamycin		NCT04461340, NCT04341675, NCT04482712	Ongoing
	Metformin (antidiabetic agent; inflammatory and immune regulator)	(+) Autophagy, Apoptosis	Retrospective study	(−) 30-day mortality
			NCT04625985	ND
			NCT04604678, NCT04510194, NCT04727424	Ongoing

(−) inhibit, (+) promote, ND not determined, c-FLIP cellular Fas-associated protein with death domain-like interleukin-1-converting enzyme-inhibitory protein, NF-κB nuclear factor kappa B, Caspase cysteine-aspartic protease, TNF-α tumor necrosis factor-alpha, RIPK receptor-interacting protein kinase, MLKL mixed lineage kinase domain-like protein, NLRP3 nucleotide-binding oligomerization domain-like receptor containing pyrin domain 3, GSDMD gasdermin-D, M^pro^ main protease, PL^pro^ papain-like protease, NSP nonstructural protein, NRF2 nuclear factor erythroid-2 related factor 2, ARDS acute respiratory distress syndrome, Ang II angiotensin II, AT1R Ang II-angiotensin 1 receptor, ACEI angiotensin-converting enzyme inhibitor, ARB angiotensin receptor blocker, PAD4 peptidylarginine deiminase 4, CXCR2 C-X-C motif chemokine receptor 2, PDE Phosphodiesterase, LDH lactate dehydrogenase, GSH glutathione, GPX4 glutathione peroxidase 4, ACSL4 acyl-CoA synthetase long-chain family member 4, ULK1 complex unc-51 like autophagy activating kinase 1 complex, PIK3C3 complex phosphatidylinositol 3-phosphate class III lipid kinase complex, mTOR mammalian target of rapamycin, AKT protein kinase B.
